# Orphenadrinium dihydrogen citrate

**DOI:** 10.1107/S1600536813001207

**Published:** 2013-01-19

**Authors:** Manpreet Kaur, Jerry P. Jasinski, Amanda C. Keeley, H. S. Yathirajan, B. P. Siddaraju

**Affiliations:** a Department of Studies in Chemistry, University of Mysore, Manasagangotri, Mysore 570 006, India; b Department of Chemistry, Keene State College, 229 Main Street, Keene, NH 03435-2001, USA; c Department of Chemistry, G. Madegowda Institute of Technology, Bharathinagara 571 442, India

## Abstract

In the title salt, C_18_H_24_NO^+^·C_6_H_7_O_7_^−^, the dihedral angle between the benzene rings in the cation is 74.2 (5)°. In the crystal, anion–anion O—H⋯O hydrogen bonds and weak O—H⋯O inter­actions form infinite chains along [100]. Between these chains, cation–anion N—H—O hydrogen bonds are observed, forming an alternate pattern of cation and anion layers and leading to a two-dimensional network parallel to (100).

## Related literature

For a clinical and pharmacological review of the efficacy of orphenadrine, see: Hunskaar & Donnel (1991 ▶). For related structures, see: Fun *et al.* (2010 ▶); Glaser *et al.* (1992 ▶); Jasinski *et al.* (2011 ▶). For standard bond lengths, see Allen *et al.* (1987 ▶).
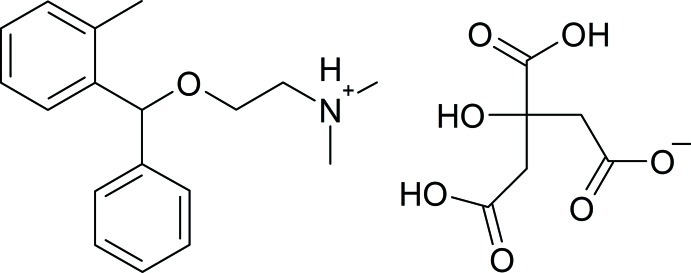


## Experimental

### 

#### Crystal data

C_18_H_24_NO^+^·C_6_H_7_O_7_^−^
*M* *_r_* = 461.50
Triclinic, 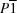

*a* = 9.9515 (8) Å
*b* = 10.7382 (9) Å
*c* = 12.625 (1) Å
α = 98.863 (7)°
β = 104.391 (7)°
γ = 111.498 (8)°
*V* = 1170.0 (2) Å^3^
*Z* = 2
Cu *K*α radiation
μ = 0.82 mm^−1^
*T* = 173 K
0.32 × 0.28 × 0.14 mm

#### Data collection

Agilent Xcalibur (Eos, Gemini) diffractometer
Absorption correction: multi-scan (*CrysAlis PRO* and *CrysAlis RED*; Agilent, 2012 ▶) *T*_min_ = 0.854, *T*_max_ = 1.000
7161 measured reflections
4471 independent reflections
3795 reflections with *I* > 2σ(*I*)
*R*_int_ = 0.029

#### Refinement

*R*[*F* ^2^ > 2σ(*F* ^2^)] = 0.056
*wR*(*F* ^2^) = 0.161
*S* = 1.03
4471 reflections
305 parameters
H-atom parameters constrained
Δρ_max_ = 0.67 e Å^−3^
Δρ_min_ = −0.27 e Å^−3^

### 

Data collection: *CrysAlis PRO* (Agilent, 2012 ▶); cell refinement: *CrysAlis PRO*; data reduction: *CrysAlis RED* (Agilent, 2012 ▶); program(s) used to solve structure: *SHELXS97* (Sheldrick, 2008 ▶); program(s) used to refine structure: *SHELXL97* (Sheldrick, 2008 ▶); molecular graphics: *SHELXTL* (Sheldrick, 2008 ▶); software used to prepare material for publication: *SHELXTL*.

## Supplementary Material

Crystal structure: contains datablock(s) global, I. DOI: 10.1107/S1600536813001207/hg5283sup1.cif

Structure factors: contains datablock(s) I. DOI: 10.1107/S1600536813001207/hg5283Isup2.hkl

Supplementary material file. DOI: 10.1107/S1600536813001207/hg5283Isup3.cml

Additional supplementary materials:  crystallographic information; 3D view; checkCIF report

## Figures and Tables

**Table 1:** Hydrogen-bond geometry (Å, °)

*D*—H⋯*A*	*D*—H	H⋯*A*	*D*⋯*A*	*D*—H⋯*A*
N1—H1⋯O6^i^	0.91	1.83	2.725 (2)	167
O4—H4*A*⋯O8^ii^	0.82	2.30	3.067 (2)	156
O7—H7*A*⋯O5^ii^	0.82	1.81	2.634 (2)	178

Symmetry codes: (i) 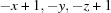; (ii) 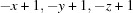.

**Fig. 1.**
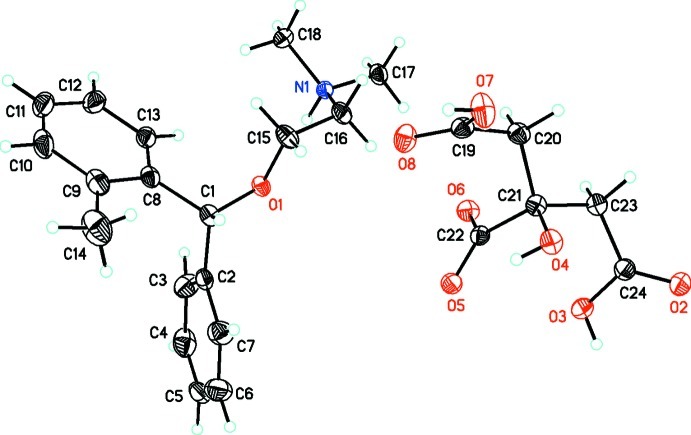
Molecular structure of the title salt showing the atom labeling scheme and 30% probability displacement ellipsoids.

**Fig. 2.**
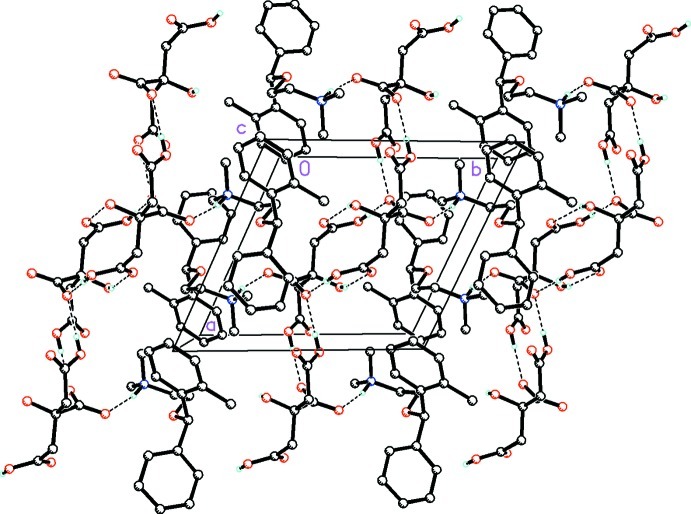
Packing diagram of the title compound viewed along the c axis. Dashed lines indicate O—H···O anion-anion hydrogen bonds and weak O—H···O intemolecular interactions in concert with cation-anion N—H···O hydrogen bonds forming an infinite two-dimensional network long [100]. The hydrogen atoms not involved in hydrogen bonding have been removed for clarity.

## supplementary crystallographic information

### Comment

Orphenadrine (systematic IUPAC name: N,N-dimethyl-2-[(2-methylphenyl) phenyl-methoxy]ethanamine) is an anticholinergic drug of the ethanolamine antihistamine class with prominent CNS and peripheral actions used to treat painful muscle spasm and other symptoms and conditions as well as some aspects of Parkinson's disease. It is closely related to diphenhydramine and therefore related to other drugs used for Parkinson's disease like benztropine and trihexyphenidyl and is also structurally related to nefopam, a centrally acting yet non-opioid analgesic. Clinical and pharmacological review of the efficacy of orphenadrine and its combination with paracetamol has been described (Hunskaar & Donnel, 1991). Orphenadrine citrate is a skeletal muscle relaxant. It acts in the central nervous system to produce its muscle relaxant effects. The orphenadrine salt used for Parkinsonism is the hydrochloride, whereas the muscle relaxant tablet is the citrate. The solid-state structure of orphenadrine hydrochloride and conformational comparisons with diphenhydramine hydrochloride and nefopam hydrochloride is reported (Glaser *et al.*, 1992). The crystal structure of orphenadrinium picrate picric acid (Fun *et al.*, 2010) and orphenadrinium picrate (Jasinski *et al.*, 2011) is recently reported. In view of the importance of orphenadrine, this paper reports the crystal structure of the title salt, (I), C_18_H_24_NO^+^. C_6_H_7_O_7_^-^.

In the title salt, C_18_H_24_NO^+^. C_6_H_7_O_7_^-^, one cation-anion pair crystallizes in the asymmetric unit (Fig. 1). The cation contains a positively charged N atom with quaternary character. The anion consists of a dihydrogen citrate counterion. The dihedral angle between the two benzene rings in the cation is 74.2 (5)°. Bond lengths are in normal ranges (Allen *et al.*, 1987). In the crystal anion-anion O—H···O hydrogen bonds and weak O—H···O intermolecular interactions form infinite chains along [100] (Table 1). In between these chains cation-anion N—H—O hydrogen bonds are observed forming an alternate pattern of cation and anion layers forming a two-dimensional network providing additional crystal stability (Fig. 2).

### Experimental

The title compound was obtained as a gift sample from R. L. Fine Chem, Bengaluru. The compound was recrystallized from methanol by slow evaporation (m. p.: 410 K).

### Refinement

All of the H atoms were placed in their calculated positions and then refined using the riding model with Atom—H lengths of 0.93Å (CH), 0.97Å (CH_2_), 0.96Å (CH_3_) 0.82Å (OH) or 0.91Å (NH). Isotropic displacement parameters for these atoms were set to 1.18-1.21 (CH, CH_2_, NH), 1.50 (CH_3_) or 1.48-1.50 (OH) times *U*_eq_ of the parent atom. The highest peak (0.67 e/A^3^) is located 0.87 Å from H4.

### Crystal data

**Table:**

C_18_H_24_NO^+^·C_6_H_7_O_7_^−^	*Z* = 2
*M**_r_* = 461.50	*F*(000) = 492
Triclinic, *P*1	*D*_x_ = 1.310 Mg m^−^^3^
Hall symbol: -P 1	Cu *K*α radiation, λ = 1.54184 Å
*a* = 9.9515 (8) Å	Cell parameters from 3067 reflections
*b* = 10.7382 (9) Å	θ = 5.1–72.4°
*c* = 12.625 (1) Å	µ = 0.82 mm^−^^1^
α = 98.863 (7)°	*T* = 173 K
β = 104.391 (7)°	Chunk, colorless
γ = 111.498 (8)°	0.32 × 0.28 × 0.14 mm
*V* = 1170.0 (2) Å^3^	

### Data collection

**Table:**

Agilent Xcalibur (Eos, Gemini) diffractometer	4471 independent reflections
Radiation source: Enhance (Cu) X-ray Source	3795 reflections with *I* > 2σ(*I*)
Graphite monochromator	*R*_int_ = 0.029
Detector resolution: 16.0416 pixels mm^-1^	θ_max_ = 72.5°, θ_min_ = 5.1°
ω scans	*h* = −11→12
Absorption correction: multi-scan (*CrysAlis PRO* and *CrysAlis RED*; Agilent, 2012)	*k* = −13→9
*T*_min_ = 0.854, *T*_max_ = 1.000	*l* = −11→15
7161 measured reflections	

### Refinement

**Table:**

Refinement on *F*^2^	Secondary atom site location: difference Fourier map
Least-squares matrix: full	Hydrogen site location: inferred from neighbouring sites
*R*[*F*^2^ > 2σ(*F*^2^)] = 0.056	H-atom parameters constrained
*wR*(*F*^2^) = 0.161	*w* = 1/[σ^2^(*F*_o_^2^) + (0.0849*P*)^2^ + 0.4888*P*] where *P* = (*F*_o_^2^ + 2*F*_c_^2^)/3
*S* = 1.03	(Δ/σ)_max_ < 0.001
4471 reflections	Δρ_max_ = 0.67 e Å^−^^3^
305 parameters	Δρ_min_ = −0.27 e Å^−^^3^
0 restraints	Extinction correction: *SHELXL97* (Sheldrick, 2008), Fc^*^=kFc[1+0.001xFc^2^λ^3^/sin(2θ)]^-1/4^
Primary atom site location: structure-invariant direct methods	Extinction coefficient: 0.0019 (7)

### Special details

**Table:**

Geometry. All esds (except the esd in the dihedral angle between two l.s. planes) are estimated using the full covariance matrix. The cell esds are taken into account individually in the estimation of esds in distances, angles and torsion angles; correlations between esds in cell parameters are only used when they are defined by crystal symmetry. An approximate (isotropic) treatment of cell esds is used for estimating esds involving l.s. planes.
Refinement. Refinement of *F*^2^ against ALL reflections. The weighted *R*-factor *wR* and goodness of fit *S* are based on *F*^2^, conventional *R*-factors *R* are based on *F*, with *F* set to zero for negative *F*^2^. The threshold expression of *F*^2^ > σ(*F*^2^) is used only for calculating *R*-factors(gt) *etc*. and is not relevant to the choice of reflections for refinement. *R*-factors based on *F*^2^ are statistically about twice as large as those based on *F*, and *R*- factors based on ALL data will be even larger.

### Fractional atomic coordinates and isotropic or equivalent isotropic displacement parameters (Å^2^)

**Table:**

	*x*	*y*	*z*	*U*_iso_*/*U*_eq_	
O1	0.37357 (15)	0.09276 (13)	0.64965 (11)	0.0291 (3)	
N1	0.22933 (17)	−0.11367 (16)	0.42955 (13)	0.0264 (3)	
H1	0.2851	−0.1302	0.4902	0.032*	
C1	0.3807 (2)	0.16847 (19)	0.75611 (16)	0.0308 (4)	
H1A	0.3877	0.2604	0.7502	0.037*	
C2	0.5265 (2)	0.1874 (2)	0.84411 (16)	0.0332 (4)	
C3	0.5437 (3)	0.0782 (3)	0.8789 (2)	0.0501 (6)	
H3	0.4637	−0.0104	0.8503	0.060*	
C4	0.6844 (3)	0.1009 (3)	0.9589 (2)	0.0575 (7)	
H4	0.6981	0.0274	0.9829	0.069*	
C5	0.7998 (3)	0.2326 (3)	1.00022 (19)	0.0528 (6)	
H5	0.8914	0.2479	1.0538	0.063*	
C6	0.7842 (3)	0.3411 (3)	0.9652 (2)	0.0582 (7)	
H6	0.8650	0.4292	0.9931	0.070*	
C7	0.6485 (3)	0.3199 (3)	0.8885 (2)	0.0491 (6)	
H7	0.6371	0.3946	0.8654	0.059*	
C8	0.2379 (2)	0.0950 (2)	0.78589 (16)	0.0328 (4)	
C9	0.1917 (3)	0.1694 (3)	0.85948 (19)	0.0458 (6)	
C10	0.0579 (3)	0.0950 (4)	0.8824 (2)	0.0572 (7)	
H10	0.0255	0.1428	0.9308	0.069*	
C11	−0.0264 (3)	−0.0451 (4)	0.8358 (2)	0.0590 (8)	
H11	−0.1147	−0.0910	0.8524	0.071*	
C12	0.0196 (3)	−0.1178 (3)	0.7648 (2)	0.0498 (6)	
H12	−0.0371	−0.2131	0.7330	0.060*	
C13	0.1520 (2)	−0.0476 (2)	0.74054 (18)	0.0366 (5)	
H13	0.1836	−0.0974	0.6929	0.044*	
C14	0.2779 (4)	0.3211 (3)	0.9121 (3)	0.0717 (9)	
H14A	0.2768	0.3689	0.8538	0.107*	
H14B	0.3817	0.3417	0.9541	0.107*	
H14C	0.2310	0.3511	0.9626	0.107*	
C15	0.2805 (3)	0.1145 (2)	0.55634 (17)	0.0366 (5)	
H15A	0.3163	0.2133	0.5625	0.044*	
H15B	0.1755	0.0790	0.5559	0.044*	
C16	0.2882 (2)	0.0405 (2)	0.44869 (17)	0.0345 (4)	
H16A	0.2293	0.0599	0.3850	0.041*	
H16B	0.3938	0.0774	0.4506	0.041*	
C17	0.2536 (2)	−0.1766 (2)	0.32629 (17)	0.0358 (5)	
H17A	0.3604	−0.1348	0.3348	0.054*	
H17B	0.1958	−0.1607	0.2608	0.054*	
H17C	0.2203	−0.2748	0.3167	0.054*	
C18	0.0653 (2)	−0.1824 (2)	0.41988 (19)	0.0390 (5)	
H18A	0.0046	−0.1606	0.3601	0.059*	
H18B	0.0529	−0.1497	0.4904	0.059*	
H18C	0.0327	−0.2813	0.4029	0.059*	
O2	0.96502 (19)	0.50720 (18)	0.23817 (14)	0.0532 (5)	
O3	0.95333 (16)	0.41174 (16)	0.38238 (13)	0.0393 (4)	
H3A	1.0444	0.4654	0.4097	0.059*	
O4	0.71300 (17)	0.54785 (13)	0.37166 (12)	0.0349 (3)	
H4A	0.7126	0.5707	0.4366	0.052*	
O5	0.74662 (15)	0.41508 (14)	0.53041 (11)	0.0331 (3)	
O6	0.63438 (16)	0.20588 (14)	0.40442 (12)	0.0346 (3)	
O7	0.3624 (2)	0.49402 (19)	0.31971 (15)	0.0492 (4)	
H7A	0.3266	0.5218	0.3652	0.074*	
O8	0.37201 (18)	0.34828 (16)	0.42549 (14)	0.0416 (4)	
C19	0.3967 (2)	0.3938 (2)	0.34734 (17)	0.0321 (4)	
C20	0.4731 (2)	0.3428 (2)	0.27215 (17)	0.0334 (4)	
H20A	0.4537	0.3712	0.2027	0.040*	
H20B	0.4292	0.2419	0.2518	0.040*	
C21	0.6483 (2)	0.40111 (19)	0.33243 (16)	0.0293 (4)	
C22	0.6792 (2)	0.33436 (19)	0.43087 (16)	0.0280 (4)	
C23	0.7190 (2)	0.3641 (2)	0.24405 (17)	0.0322 (4)	
H23A	0.6873	0.2643	0.2233	0.039*	
H23B	0.6799	0.3893	0.1762	0.039*	
C24	0.8912 (2)	0.4362 (2)	0.28604 (17)	0.0342 (4)	

### Atomic displacement parameters (Å^2^)

**Table:**

	*U*^11^	*U*^22^	*U*^33^	*U*^12^	*U*^13^	*U*^23^
O1	0.0349 (7)	0.0325 (7)	0.0252 (7)	0.0184 (6)	0.0105 (5)	0.0109 (5)
N1	0.0263 (8)	0.0323 (8)	0.0251 (7)	0.0157 (6)	0.0095 (6)	0.0101 (6)
C1	0.0404 (11)	0.0278 (9)	0.0283 (9)	0.0180 (8)	0.0118 (8)	0.0090 (7)
C2	0.0361 (10)	0.0412 (11)	0.0253 (9)	0.0174 (9)	0.0129 (8)	0.0102 (8)
C3	0.0409 (12)	0.0553 (14)	0.0614 (15)	0.0224 (11)	0.0167 (11)	0.0313 (12)
C4	0.0664 (17)	0.0791 (19)	0.0546 (15)	0.0466 (16)	0.0286 (13)	0.0389 (14)
C5	0.0428 (13)	0.0832 (19)	0.0266 (10)	0.0263 (13)	0.0084 (9)	0.0053 (11)
C6	0.0465 (14)	0.0612 (16)	0.0493 (14)	0.0168 (12)	0.0071 (11)	−0.0025 (12)
C7	0.0484 (13)	0.0443 (12)	0.0469 (13)	0.0179 (11)	0.0130 (11)	0.0005 (10)
C8	0.0368 (10)	0.0464 (11)	0.0268 (9)	0.0272 (9)	0.0116 (8)	0.0150 (8)
C9	0.0537 (14)	0.0657 (15)	0.0340 (11)	0.0427 (12)	0.0143 (10)	0.0136 (10)
C10	0.0616 (16)	0.104 (2)	0.0393 (12)	0.0599 (17)	0.0272 (12)	0.0284 (14)
C11	0.0410 (13)	0.102 (2)	0.0510 (15)	0.0371 (15)	0.0231 (12)	0.0378 (16)
C12	0.0377 (12)	0.0678 (16)	0.0487 (13)	0.0217 (11)	0.0155 (10)	0.0285 (12)
C13	0.0363 (10)	0.0476 (12)	0.0336 (10)	0.0219 (9)	0.0132 (8)	0.0190 (9)
C14	0.096 (2)	0.074 (2)	0.0606 (17)	0.0541 (19)	0.0309 (17)	0.0065 (15)
C15	0.0526 (12)	0.0351 (10)	0.0280 (10)	0.0257 (10)	0.0091 (9)	0.0133 (8)
C16	0.0440 (11)	0.0340 (10)	0.0281 (9)	0.0177 (9)	0.0105 (8)	0.0149 (8)
C17	0.0399 (11)	0.0468 (12)	0.0285 (10)	0.0242 (9)	0.0151 (8)	0.0102 (8)
C18	0.0274 (10)	0.0494 (12)	0.0383 (11)	0.0145 (9)	0.0120 (8)	0.0093 (9)
O2	0.0459 (9)	0.0542 (10)	0.0379 (9)	−0.0012 (8)	0.0148 (7)	0.0088 (7)
O3	0.0296 (7)	0.0457 (8)	0.0417 (8)	0.0149 (6)	0.0121 (6)	0.0121 (7)
O4	0.0454 (8)	0.0265 (7)	0.0360 (7)	0.0169 (6)	0.0155 (6)	0.0095 (6)
O5	0.0300 (7)	0.0403 (8)	0.0317 (7)	0.0180 (6)	0.0104 (6)	0.0092 (6)
O6	0.0375 (8)	0.0299 (7)	0.0388 (8)	0.0170 (6)	0.0096 (6)	0.0137 (6)
O7	0.0714 (11)	0.0648 (11)	0.0521 (10)	0.0544 (10)	0.0361 (9)	0.0359 (9)
O8	0.0487 (9)	0.0396 (8)	0.0515 (9)	0.0239 (7)	0.0257 (7)	0.0246 (7)
C19	0.0279 (9)	0.0339 (10)	0.0366 (10)	0.0155 (8)	0.0083 (8)	0.0134 (8)
C20	0.0347 (10)	0.0382 (10)	0.0322 (10)	0.0212 (9)	0.0088 (8)	0.0121 (8)
C21	0.0321 (10)	0.0267 (9)	0.0329 (10)	0.0151 (8)	0.0114 (8)	0.0108 (7)
C22	0.0249 (9)	0.0325 (9)	0.0323 (10)	0.0159 (8)	0.0108 (7)	0.0122 (8)
C23	0.0347 (10)	0.0313 (9)	0.0324 (10)	0.0156 (8)	0.0120 (8)	0.0083 (8)
C24	0.0368 (10)	0.0307 (9)	0.0313 (10)	0.0116 (8)	0.0133 (8)	0.0016 (8)

### Geometric parameters (Å, º)

**Table:**

O1—C15	1.415 (2)	C14—H14B	0.9600
O1—C1	1.431 (2)	C14—H14C	0.9600
N1—C18	1.488 (2)	C15—C16	1.500 (3)
N1—C17	1.490 (2)	C15—H15A	0.9700
N1—C16	1.496 (2)	C15—H15B	0.9700
N1—H1	0.9100	C16—H16A	0.9700
C1—C2	1.514 (3)	C16—H16B	0.9700
C1—C8	1.522 (3)	C17—H17A	0.9600
C1—H1A	0.9800	C17—H17B	0.9600
C2—C3	1.366 (3)	C17—H17C	0.9600
C2—C7	1.400 (3)	C18—H18A	0.9600
C3—C4	1.418 (4)	C18—H18B	0.9600
C3—H3	0.9300	C18—H18C	0.9600
C4—C5	1.369 (4)	O2—C24	1.200 (3)
C4—H4	0.9300	O3—C24	1.332 (3)
C5—C6	1.354 (4)	O3—H3A	0.8200
C5—H5	0.9300	O4—C21	1.414 (2)
C6—C7	1.367 (4)	O4—H4A	0.8200
C6—H6	0.9300	O5—C22	1.265 (2)
C7—H7	0.9300	O6—C22	1.244 (2)
C8—C13	1.389 (3)	O7—C19	1.313 (2)
C8—C9	1.403 (3)	O7—H7A	0.8200
C9—C10	1.406 (4)	O8—C19	1.206 (2)
C9—C14	1.480 (4)	C19—C20	1.511 (3)
C10—C11	1.368 (4)	C20—C21	1.550 (3)
C10—H10	0.9300	C20—H20A	0.9700
C11—C12	1.368 (4)	C20—H20B	0.9700
C11—H11	0.9300	C21—C23	1.535 (3)
C12—C13	1.394 (3)	C21—C22	1.549 (3)
C12—H12	0.9300	C23—C24	1.505 (3)
C13—H13	0.9300	C23—H23A	0.9700
C14—H14A	0.9600	C23—H23B	0.9700
			
C15—O1—C1	112.03 (14)	O1—C15—C16	108.73 (16)
C18—N1—C17	109.99 (15)	O1—C15—H15A	109.9
C18—N1—C16	113.44 (15)	C16—C15—H15A	109.9
C17—N1—C16	109.95 (15)	O1—C15—H15B	109.9
C18—N1—H1	107.8	C16—C15—H15B	109.9
C17—N1—H1	107.8	H15A—C15—H15B	108.3
C16—N1—H1	107.8	N1—C16—C15	113.81 (16)
O1—C1—C2	107.13 (15)	N1—C16—H16A	108.8
O1—C1—C8	111.31 (16)	C15—C16—H16A	108.8
C2—C1—C8	112.95 (15)	N1—C16—H16B	108.8
O1—C1—H1A	108.4	C15—C16—H16B	108.8
C2—C1—H1A	108.4	H16A—C16—H16B	107.7
C8—C1—H1A	108.4	N1—C17—H17A	109.5
C3—C2—C7	119.2 (2)	N1—C17—H17B	109.5
C3—C2—C1	121.8 (2)	H17A—C17—H17B	109.5
C7—C2—C1	119.03 (19)	N1—C17—H17C	109.5
C2—C3—C4	119.5 (2)	H17A—C17—H17C	109.5
C2—C3—H3	120.2	H17B—C17—H17C	109.5
C4—C3—H3	120.2	N1—C18—H18A	109.5
C5—C4—C3	119.0 (2)	N1—C18—H18B	109.5
C5—C4—H4	120.5	H18A—C18—H18B	109.5
C3—C4—H4	120.5	N1—C18—H18C	109.5
C6—C5—C4	121.8 (2)	H18A—C18—H18C	109.5
C6—C5—H5	119.1	H18B—C18—H18C	109.5
C4—C5—H5	119.1	C24—O3—H3A	109.5
C5—C6—C7	119.4 (3)	C21—O4—H4A	109.5
C5—C6—H6	120.3	C19—O7—H7A	109.5
C7—C6—H6	120.3	O8—C19—O7	123.69 (19)
C6—C7—C2	121.1 (2)	O8—C19—C20	123.37 (17)
C6—C7—H7	119.5	O7—C19—C20	112.93 (17)
C2—C7—H7	119.5	C19—C20—C21	111.50 (16)
C13—C8—C9	119.1 (2)	C19—C20—H20A	109.3
C13—C8—C1	120.07 (17)	C21—C20—H20A	109.3
C9—C8—C1	120.8 (2)	C19—C20—H20B	109.3
C8—C9—C10	117.7 (2)	C21—C20—H20B	109.3
C8—C9—C14	122.4 (2)	H20A—C20—H20B	108.0
C10—C9—C14	119.9 (2)	O4—C21—C23	107.28 (15)
C11—C10—C9	122.3 (2)	O4—C21—C22	111.65 (15)
C11—C10—H10	118.8	C23—C21—C22	110.56 (15)
C9—C10—H10	118.8	O4—C21—C20	110.38 (14)
C12—C11—C10	119.9 (2)	C23—C21—C20	107.75 (16)
C12—C11—H11	120.1	C22—C21—C20	109.14 (15)
C10—C11—H11	120.1	O6—C22—O5	126.11 (17)
C11—C12—C13	119.4 (3)	O6—C22—C21	116.70 (16)
C11—C12—H12	120.3	O5—C22—C21	117.19 (16)
C13—C12—H12	120.3	C24—C23—C21	113.05 (16)
C8—C13—C12	121.5 (2)	C24—C23—H23A	109.0
C8—C13—H13	119.2	C21—C23—H23A	109.0
C12—C13—H13	119.2	C24—C23—H23B	109.0
C9—C14—H14A	109.5	C21—C23—H23B	109.0
C9—C14—H14B	109.5	H23A—C23—H23B	107.8
H14A—C14—H14B	109.5	O2—C24—O3	123.4 (2)
C9—C14—H14C	109.5	O2—C24—C23	124.1 (2)
H14A—C14—H14C	109.5	O3—C24—C23	112.52 (17)
H14B—C14—H14C	109.5		
			
C15—O1—C1—C2	−156.91 (16)	C10—C11—C12—C13	−0.2 (4)
C15—O1—C1—C8	79.15 (19)	C9—C8—C13—C12	1.2 (3)
O1—C1—C2—C3	−69.6 (2)	C1—C8—C13—C12	−179.11 (19)
C8—C1—C2—C3	53.3 (3)	C11—C12—C13—C8	−0.7 (3)
O1—C1—C2—C7	108.4 (2)	C1—O1—C15—C16	175.89 (16)
C8—C1—C2—C7	−128.6 (2)	C18—N1—C16—C15	61.1 (2)
C7—C2—C3—C4	−0.1 (4)	C17—N1—C16—C15	−175.25 (17)
C1—C2—C3—C4	178.0 (2)	O1—C15—C16—N1	61.9 (2)
C2—C3—C4—C5	0.5 (4)	O8—C19—C20—C21	−76.4 (2)
C3—C4—C5—C6	−1.3 (4)	O7—C19—C20—C21	102.6 (2)
C4—C5—C6—C7	1.6 (4)	C19—C20—C21—O4	−54.0 (2)
C5—C6—C7—C2	−1.1 (4)	C19—C20—C21—C23	−170.86 (15)
C3—C2—C7—C6	0.4 (4)	C19—C20—C21—C22	69.05 (19)
C1—C2—C7—C6	−177.8 (2)	O4—C21—C22—O6	−176.07 (15)
O1—C1—C8—C13	24.5 (2)	C23—C21—C22—O6	−56.7 (2)
C2—C1—C8—C13	−96.0 (2)	C20—C21—C22—O6	61.6 (2)
O1—C1—C8—C9	−155.81 (17)	O4—C21—C22—O5	4.3 (2)
C2—C1—C8—C9	83.6 (2)	C23—C21—C22—O5	123.66 (17)
C13—C8—C9—C10	−0.9 (3)	C20—C21—C22—O5	−117.99 (18)
C1—C8—C9—C10	179.40 (18)	O4—C21—C23—C24	51.0 (2)
C13—C8—C9—C14	179.4 (2)	C22—C21—C23—C24	−71.0 (2)
C1—C8—C9—C14	−0.3 (3)	C20—C21—C23—C24	169.85 (16)
C8—C9—C10—C11	0.1 (3)	C21—C23—C24—O2	−124.6 (2)
C14—C9—C10—C11	179.8 (2)	C21—C23—C24—O3	55.7 (2)
C9—C10—C11—C12	0.4 (4)		

### Hydrogen-bond geometry (Å, º)

**Table:**

*D*—H···*A*	*D*—H	H···*A*	*D*···*A*	*D*—H···*A*
N1—H1···O6^i^	0.91	1.83	2.725 (2)	167
O4—H4*A*···O8^ii^	0.82	2.30	3.067 (2)	156
O7—H7*A*···O5^ii^	0.82	1.81	2.634 (2)	178

Symmetry codes: (i) −*x*+1, −*y*, −*z*+1; (ii) −*x*+1, −*y*+1, −*z*+1.
